# Visual duration bisection in profoundly deaf individuals

**DOI:** 10.7717/peerj.10133

**Published:** 2020-10-20

**Authors:** Feng Zhang, Kaige Jin, Sainan Zhang

**Affiliations:** Institute of Psychology and Behavior, Henan University, Kaifeng, Henan, China

**Keywords:** Deaf, Duration bisection, Temporal perception, Vision

## Abstract

**Background:**

Previous research has been designed to study the effect of hearing loss on supra-second duration estimation in the visual channel and position effect of visual abilities among deaf populations. The current study aimed to investigate the sub-second duration perception of different visual fields in profoundly deaf individuals.

**Methods:**

A total of 16 profoundly deaf undergraduates and 16 hearing undergraduates completed a visual duration bisection task in which participants made judgments about whether a series of probe durations that were linearly spaced from 200 ms to 800 ms at 100 ms intervals were more similar to a standard short duration (200 ms) or a standard long duration (800 ms). The probe stimuli were presented in the center, left, or right of the screen. A repeated measure analysis of variance (ANOVA) with a between-participants factor of group and a within-participants factor of position, and a one-sample *t*-test were conducted.

**Results:**

The Weber ratio (WR) values of deaf participants were significantly higher than those of hearing participants, regardless of the presented positions of the visual stimulus. The bisection point (BP) value of deaf participants was significantly lower than 500 ms (average mean of 200/800 ms) and the BP value of hearing participants did not significantly differ from 500 ms, although the overall difference of BP values between the deaf group and hearing group did not reach significance. For deaf participants, the BP value in the center condition was significantly lower than 500 ms; however, the difference between the BP value in the left condition and 500 ms did not reach significance, indicating that their duration discrimination accuracy in the left visual field was better than that in the center visual field.

**Conclusions:**

Hearing loss impaired visual sub-second duration perception, and deaf individuals showed a left visual field advantage of duration discrimination accuracy during the visual duration bisection task.

## Introduction

Compared with the visual system, the auditory system plays a more important role in temporal perception. It was found that auditory rhythms were reproduced more accurately than visual rhythms, which showed that the coding of time was more accurate for auditory events than it was for visual events (Glenberg & Jona, 1991). As an inherent temporal signal, sound can provide a perceptual scaffolding for temporal behavior (Conway, Pisoni & Kronenberger, 2009). Therefore, the loss of auditory experience can affect the normal development of temporal perception (Gori, Sandini & Burr, 2012), and can cause the deaf individuals psychological distress (Cheng, Chou & Lin, 2019), which was related to loneliness and self-esteem (Mamun et al., 2020) and which needed the promotion of personal and population resilience (Ivbijaro et al., 2019).

The existing research has demonstrated the generalized-deficiency hypothesis proposed by Myklebust (1964). Deafness is a physical impairment associated with functional disability and an auditory deficit that affects the neurological development and organization of other perceptual systems. This idea suggests that hearing loss may reduce the perception abilities of other senses. The evidence showed that hearing loss impaired visual temporal perception of deaf individuals (Bolognini et al., 2012; Heming & Brown, 2005; Kowalska & Szelag, 2006). Kowalska & Szelag (2006) employed temporal estimation method and temporal reproduction method to investigate the effect of congenital deafness on the durations of visual stimuli, and found that the deaf individuals overestimated significantly the shorter durations (under 2 s) and underestimated significantly the longer durations (above 3 s), showing poorer duration judgment accuracies than hearing ones. Bolognini et al. (2012) explored the effect of hearing loss on duration discrimination of tactile stimuli in deaf individuals, and the results showed that the duration perceptual sensitivity was significantly poorer in deaf individuals than in hearing controls.

However, some research results have demonstrated a compensatory effect due to the auditory cortex subserving visual functions in deaf individuals (Pavani & Bottari, 2012). Nava et al. (2008) did not find significant differences between 12 deaf individuals and 10 hearing individuals in visual temporal order judgment (TOJ). In their experiment, the stimulus onset asynchronies were 20 ms, 30 ms, 55 ms, 90 ms, or 110 ms, and the participants were instructed to make judgments on which visual target appeared first, resulting in no differences in proportions of correct responses, point of subjective simultaneity, and just noticeable difference (JND). Poizner & Tallal (1987) observed similar results in critical flicker frequency threshold or two-point threshold of visual stimuli indicating that deaf individuals did not have any deficits of simultaneity and temporal order in the visual perception.

Moreover, the findings of previous studies have shown that there has a significant spatial position effect of visual abilities following deafness. Compared to hearing controls, deaf individuals had a priority in processing peripheral stimuli (Proksch & Bavelier, 2002) and faster discrimination responses were observed in deaf individuals especially when the first stimulus appeared at peripheral positions in visual TOJs (Nava et al., 2008). In addition, visual attention to the periphery was enhanced in congenitally deaf individuals (Bavelier et al., 2000) and peripheral stimuli produced significantly higher visual evoked potentials than in hearing populations (Neville, Schmidt & Kutas, 1983). In contrast, however, the results from simultaneity judgment task did not find the spatial position difference (Heming & Brown, 2005). In this last study, six light emitting diodes (LED) were symmetrically arranged with respect to the center of the visual display, and there were no significant position effects and no left hemisphere (LH)/right visual field (RVF) advantage when deaf individuals perceived the onset of the visual stimulus pairs as simultaneous or non-simultaneous. Nevertheless, one aspect that remains to be ascertained is whether the spatial position effect on temporal order perception extends to visual duration perception.

In terms of visual duration perception in deaf individuals, although Kowalska & Szelag (2006) have explored it within supra-second range, the sub-second perception has not been investigated. Given the controversial findings of visual position effects on temporal processing in deaf individuals (Heming & Brown, 2005; Nava et al., 2008), it may be advantageous to examine duration perception within sub-second range from a lateralized perspective. Moreover, the duration bisection paradigm is a classic task employed to examine how humans perceive time (Ng, Tobin & Penney, 2011). Therefore, in the current work, a visual duration bisection task with seven probe durations that were linearly spaced from 200 to 800 ms at 100 ms intervals (i.e., 200, 300, 400, 500, 600, 700, and 800 ms) was used with a 2 (group: deaf vs. hearing) × 3 (position: center, left, and right of the screen) mixed design to explore the effect of hearing loss on sub-second duration perception.

## Materials and Methods

### Participants

A total of 32 right-handed undergraduates volunteered for this study. Sixteen profoundly deaf undergraduates (3 females and 13 males) whose mean age was 20.19 years (SD = 0.98) ranging from 18 to 21 years took part in the study, and they all had bilateral profound hearing loss (>85 dB). Sixteen hearing undergraduates (13 females and 3 males) whose mean age was 19.56 years (SD = 0.89) ranging from 18 to 21 years also participated in the study. There was no significant difference in age between the deaf group and the hearing group, *t* (30) = −1.89, *p* = 0.069.

Both deaf and hearing undergraduates reported good physical health and had normal or corrected-to-normal vision. The experimental protocol was approved by Ethics Committee of Henan University in China (HU2018-192 and 20181103). All participants gave their written informed consent before the study, and they received payment after the experiment.

### Experimental stimuli and procedure

The visual stimulus consisted of a 3 cm square presented on the left, right, or center of the visual display and they were equally spaced at 8 cm (center to center) on a horizontal plane. Participants were tested at a distance of 60 cm from the screen in a quiet room. Deaf participants were provided with written instructions and their questions were answered by a sign language teacher. All participants were asked to keep their eyes on the screen during the entire process of the experiment.

During the duration bisection task, the short standard duration was 200 ms, the long standard duration was 800 ms, and the probe durations were 200, 300, 400, 500, 600, 700, and 800 ms. In the beginning, the two standard durations were presented on the screen center five times each, and participants were told that it was the long standard duration or the short standard duration. Then, participants were trained to discriminate between short and long standard durations ten times and were given visual feedback showing correct or wrong answer. Third, on each trial of the duration bisection task being administered for one of the seven probe durations, participants were asked to judge whether the presented duration was more similar to the short standard duration or to the long standard duration by pressing one of two keys (D or K) when the probe stimulus disappeared from the screen, and the inter-trial interval was 800 ms. The entire experiment lasted about 25 min.

### Statistical analysis

Visual duration perception was analyzed by calculating the bisection point (BP) and Weber ratio (WR) obtained from the deaf and hearing individual psychometric functions which were represented by the proportion of long responses against the probe durations in the different positions.

The BP is the stimulus duration of 50% “long” responses. This measure was derived from the slope and intercept parameters obtained by fitting a logistic function to the individual data (Droit-Volet, Fayolle & Gil, 2011). The BP value in humans was close to the arithmetic mean (AM) of the standard short and long durations when the probe durations were spaced linearly (Wearden, 1991; Wearden & Ferrara, 1995), and in the present study with durations being spaced in equal linear (100 ms) steps, the AM of 200/800 ms was 500 ms. WR is an index of temporal sensitivity, whose value is the Difference Limen (DL) divided by the BP (Droit-Volet, Fayolle & Gil, 2011). A lower BP value indicates that durations are overestimated, and a lower WR value means greater sensitivity to time.

The BP and WR values were analyzed using a repeated measure analysis of variance (ANOVA) with a between-participants factor of group (deaf or hearing) and a within-participants factor of position (center, left, or right). A one-sample *t*-test was conducted to determine whether BP values differed from 500 ms (AM of 200 ms and 800 ms) in different conditions. All statistical analyses used an α level of 0.05.

## Results

**Figure 1:**
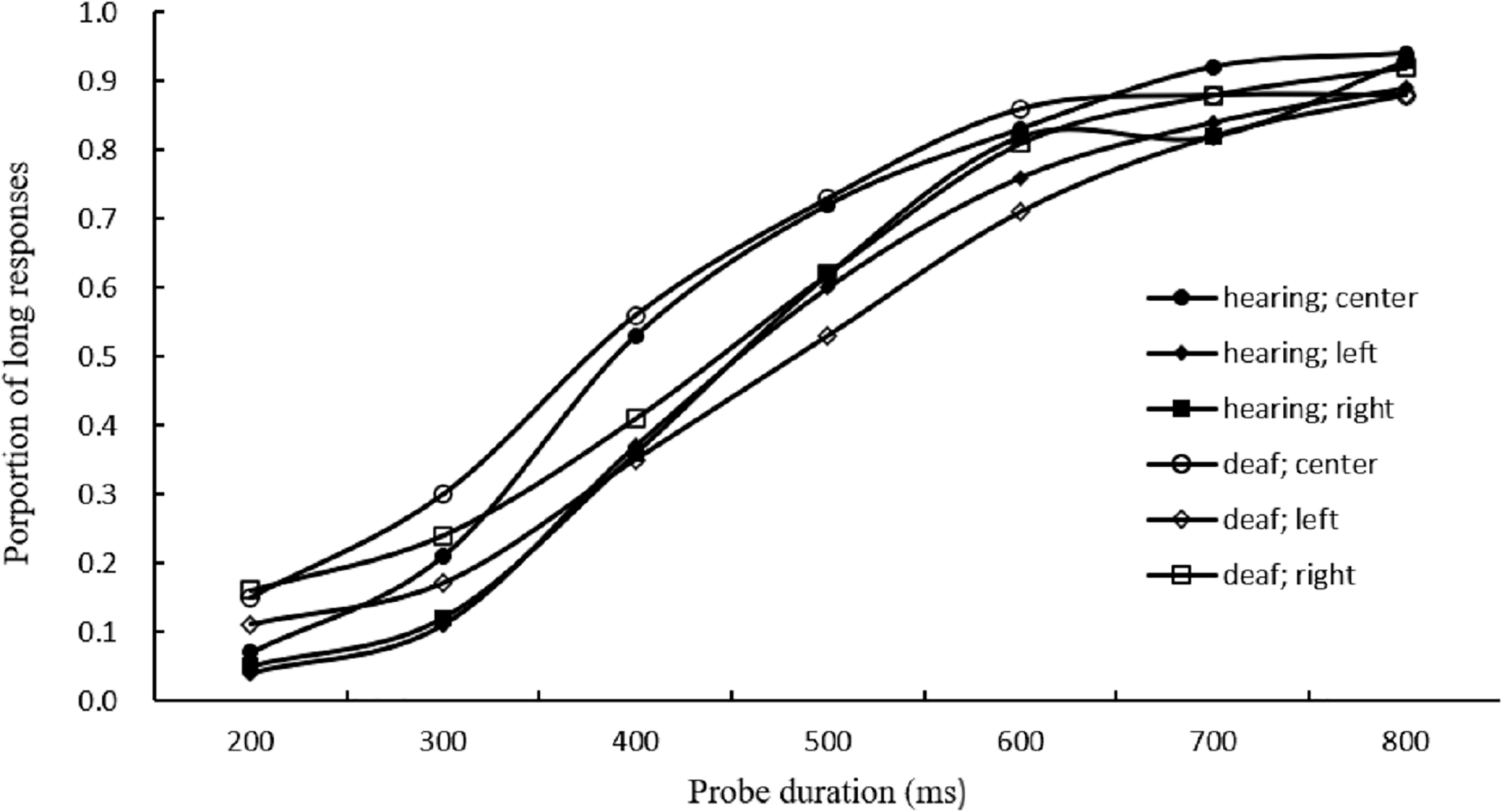
Results for long responses. Average proportion of long responses in visual duration bisection task for the hearing and deaf groups.

Figure 1 showed the average proportion of long responses for each condition.

### Bisection point

**Figure 2:**
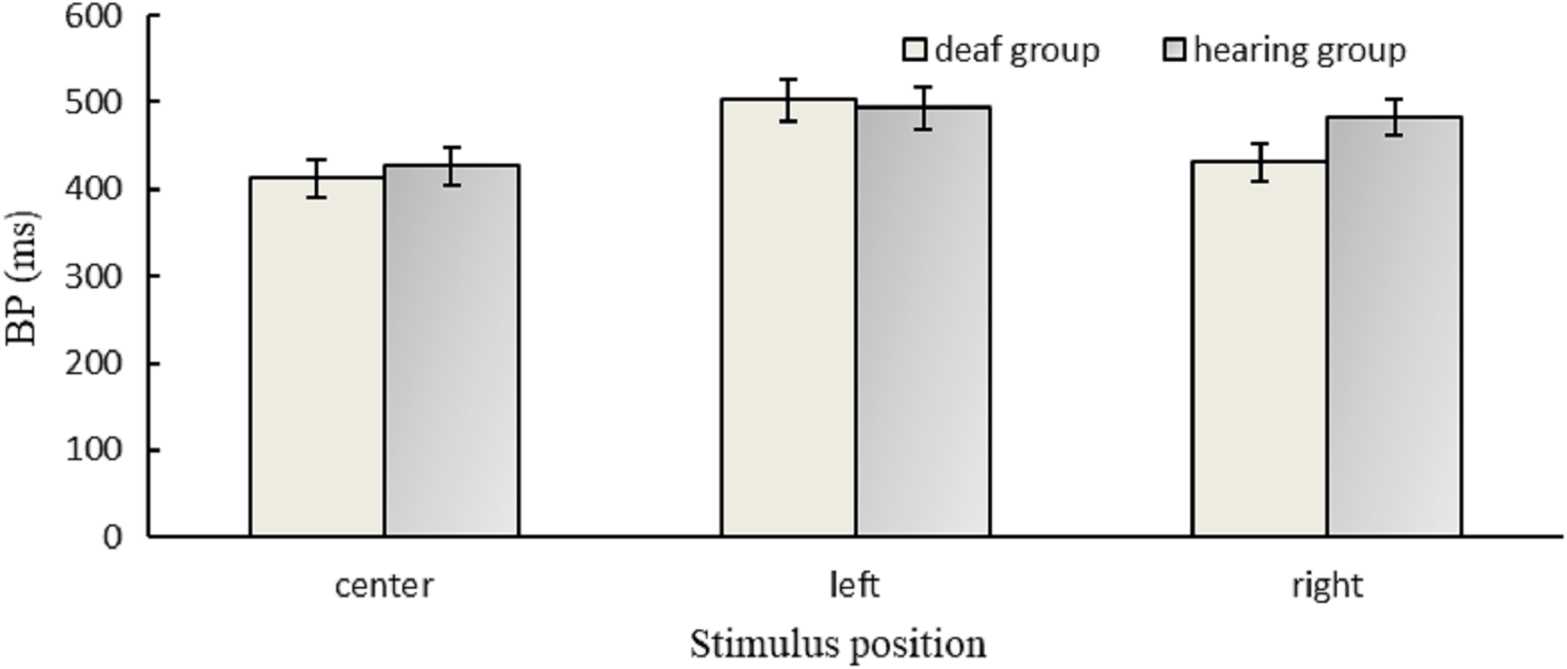
Results for BP. BP values in different positions for the hearing and deaf groups.

Figure 2 presented the BP values for different conditions. A larger BP value means that the durations are underestimated.

The ANOVA of BP indicated that there was a significant main effect of position, *F*(2,60) = 16.61, *p* < 0.001, *η*^2^ = 0.36. The results of the post hoc test/Least Significant Difference (LSD) showed that the BP value of the left (498.13 ms) was higher than the right (456.84 ms), *p* = 0.004, and both the BP values of the left and the right were higher than the center (419.27 ms), *p*s <0.05. The BP value of deaf participants (448.56 ms) was not different from that of hearing participants (467.60 ms), *F*(1,30) = 0.47, *p* = 0.500. There was no significant interaction between group and position, *F*(2,60) = 2.52, *p* = 0.089.

A one-sample *t*-test was performed to compare the BP value of each group with 500 ms (AM of 200/800 ms). The results showed that the BP value of the deaf group was significantly lower than 500 ms, *t* (15) = −3.22, *p* = 0.006; however, there was no significant difference between the BP value of the hearing group and 500 ms, *t* (15) =-1.44, *p* = 0.172, suggesting that deaf individuals overestimated durations; but hearing individuals did not.

To further test the positional effect for deaf participants, a one-sample *t*-test indicated that there was a significant difference between the BP value (419.27 ms) in the center condition and 500 ms, *t* (15) = −4.80, *p* < 0.001; however, the difference between the BP value (502.57 ms) in the left condition and 500 ms did not reach significance, *t* (15) = 0.11, *p* = 0.917, indicating the accuracy of duration discrimination in the left visual field was better than that in the center of their visual field for deaf individuals.

### Weber ratio

**Figure 3:**
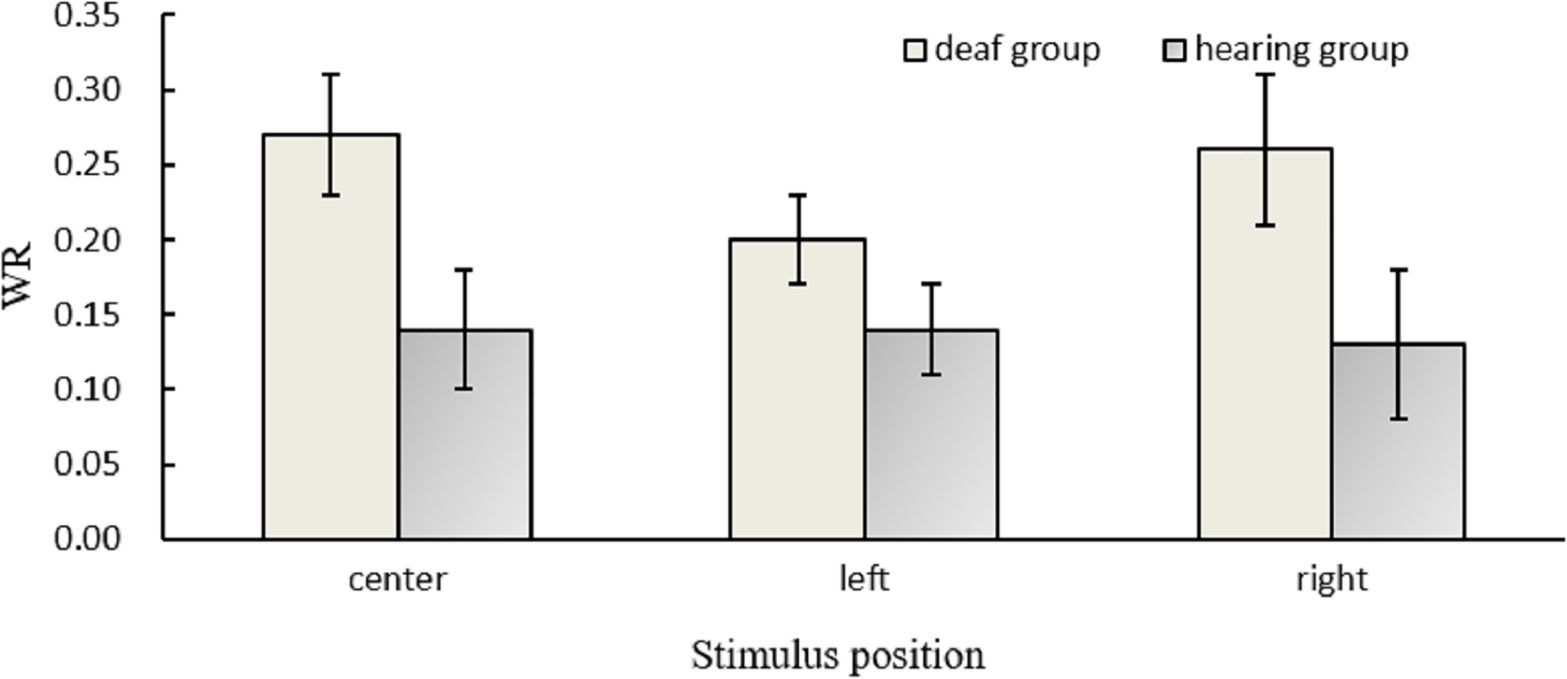
Results for WR. Temporal sensitivity (WR values) in different positions for the hearing and deaf groups.

Figure 3 presented the WR values for different conditions. A higher WR value means that it is more difficult to discriminate between two durations.

The ANOVA of WR suggested that there was a significant main effect of the group, *F*(1,30) = 5.15, *p* = 0.031, *η*^2^ = 0.15, indicating that the WR value of deaf participants (0.24) was higher than that of hearing participants (0.14). However, the main effect of position and the interaction between group and position did not reach significance, *p*s > 0.05.

For deaf participants, there was no significant position effect, *F* (2, 45) = 0.42, *p* = 0.660, indicating that their poor duration discrimination sensitivity had no hemisphere difference or positional effect.

## Discussion

The current study examined whether hearing loss can affect sub-second perception using visual duration bisection task in profoundly deaf individuals. BP is an index of perceived duration which is considered as temporal bias toward responding “long” or “short” (Droit-Volet, Fayolle & Gil, 2011), and our study results found that deaf individuals overestimated significantly the presented durations; but hearing individuals did not, although overall the BP values between the deaf group and hearing group did not differ significantly. With respect to temporal sensitivity (WR values), our results demonstrated that compared to hearing individuals, deaf individuals had higher WR values during the measurement of sub-second regardless of the spatial position in which the visual stimulus was located on the left, right, or center of the screen. In other words, deaf individuals found it more difficult to discriminate the probe durations than hearing individuals. In brief, our study findings indicated that the deaf were impaired in visual duration perception, which was in line with the discrimination of the temporal duration of touches from Bolognini et al. (2012) and supra-second temporal estimation of visual channels from Kowalska & Szelag (2006). Our study which focused on the categorization of sub-second duration by means of a bisection task expanded the current research results (Bolognini et al., 2012; Kowalska & Szelag, 2006), and added new supporting evidence of the generalized-deficiency hypothesis (Myklebust, 1964).

These study results also illustrated that there was a significant position effect on the BP values. The BP is the point of subjective equality and a decrease in the BP value means a lengthening effect (Droit-Volet, Fayolle & Gil, 2011). For deaf participants in the present study, the BP value in the center condition was significantly lower than 500 ms; however, there was no significant difference between the BP value in the left condition and 500 ms, indicating that the accuracy of duration judgment of the center condition was significantly poorer than that of the left condition. This was in accordance with the faster reactivity to the two peripheral stimuli in the visual TOJ in deaf participants (Nava et al., 2008), which demonstrated that the deaf individuals had a greater need to use peripheral vision for monitoring their surroundings.

However, contrary to the RVF/LH advantage for deaf populations (Bosworth & Dobkins, 1999), the lateralization finding of the current study confirmed the left visual field (LVF) advantage suggesting better duration discrimination performance in the LVF compared to in the center field of deaf participants. Given that evidence has confirmed the existence of a RVF temporal-processing advantage (Corballis, 1996; Efron, 1963; Mills & Rollman, 1980; Nicholls & Lindell, 2000) and our study result that the deaf no longer had the RVF advantage to discriminate sub-second durations, it could be inferred that hearing loss impaired temporal duration perception.

In comparison with other reported temporal perception in the sub-second range, compared with the control group, the higher WR value (0.24) for the deaf group in this study was comparable to the higher visual temporal threshold (68.93 ms) for the deaf group in Heming & Brown (2005) findings. However, the deaf group did not reveal significant differences in temporal order perception of rapidly changing visual forms in comparison to the hearing group (Poizner & Tallal, 1987) and the JND values showed no significant difference between the deaf and hearing individuals (Nava et al., 2008). Therefore, the available evidence suggested that the effect of hearing loss on temporal perception may depend on the type of temporal discrimination task, which should be explored in a future study.

## Conclusions

In conclusion, unlike previous studies focusing on visual supra-second duration perception of deaf individuals, the current study found impaired sub-second duration perception in visual channel of deaf individuals. Deaf participants had higher WR values than hearing ones. The accuracy of duration discrimination in the left visual field was better than in the center visual field for deaf individuals suggesting that they showed a LVF advantage during the visual duration bisection task.

## Supplemental Information

10.7717/peerj.10133/supp-1 Supplemental Information 1 Long responses in the conditions of different positions and probe durations of visual stimulus for each participant. The data in each row indicates the response (long or short) to one of seven probe durations and one of three positions in every trial.

10.7717/peerj.10133/supp-2 Supplemental Information 2 The basic information of participants. The data in a row indicates the basic information including sex, age, and group for each participant.

## Additional Information and Declarations

### Data Availability

The following information was supplied regarding data availability: The raw measurements are available in the Supplemental Files.
